# Supplementary material for: Stroma-derived Dickkopf-1 contributes to the suppression of NK cell cytotoxicity in breast cancer
Source: Nat Commun. 2025 Jan 30;16:1183. doi: 10.1038/s41467-025-56420-w (PMC11782527; doi:10.1038/s41467-025-56420-w)
Supplement: Supplementary file 1 — Supplementary Information [file 41467_2025_56420_MOESM1_ESM.pdf]

Supplementary Figure 1.

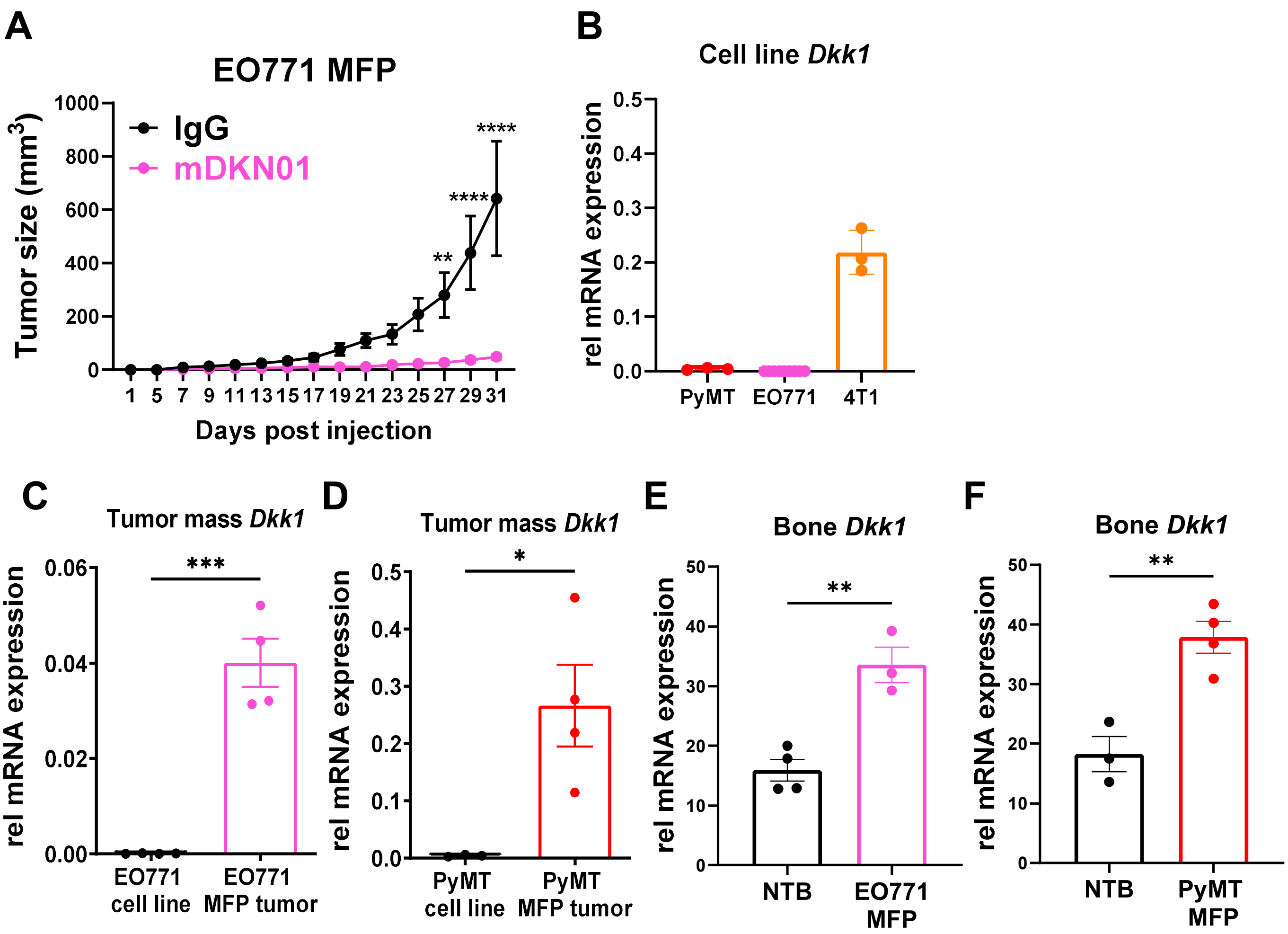

(A) WT mice were inoculated with EO771 (n=4 mice/group) and received mDKN01 (10mg/kg) or control IgG antibody i.p. every other day. Tumor growth was determined by caliper measurements. (B-F) *Dkk1* expression was measured in tumor cell lines (B), the primary tumors (C, D), and bones of no tumor (NTB) and tumor bearing mice (E, F). Experiments in (B) were performed in triplicate. Two-way ANOVA followed by Bonferroni multiple-comparison test (A), and unpaired t-test with two-tailed P value for (C-F) were used to determine significance * $P < 0.05$, ** $P < 0.01$, *** $P < 0.001$. **** $P < 0.0001$.

Supplementary Figure 2.

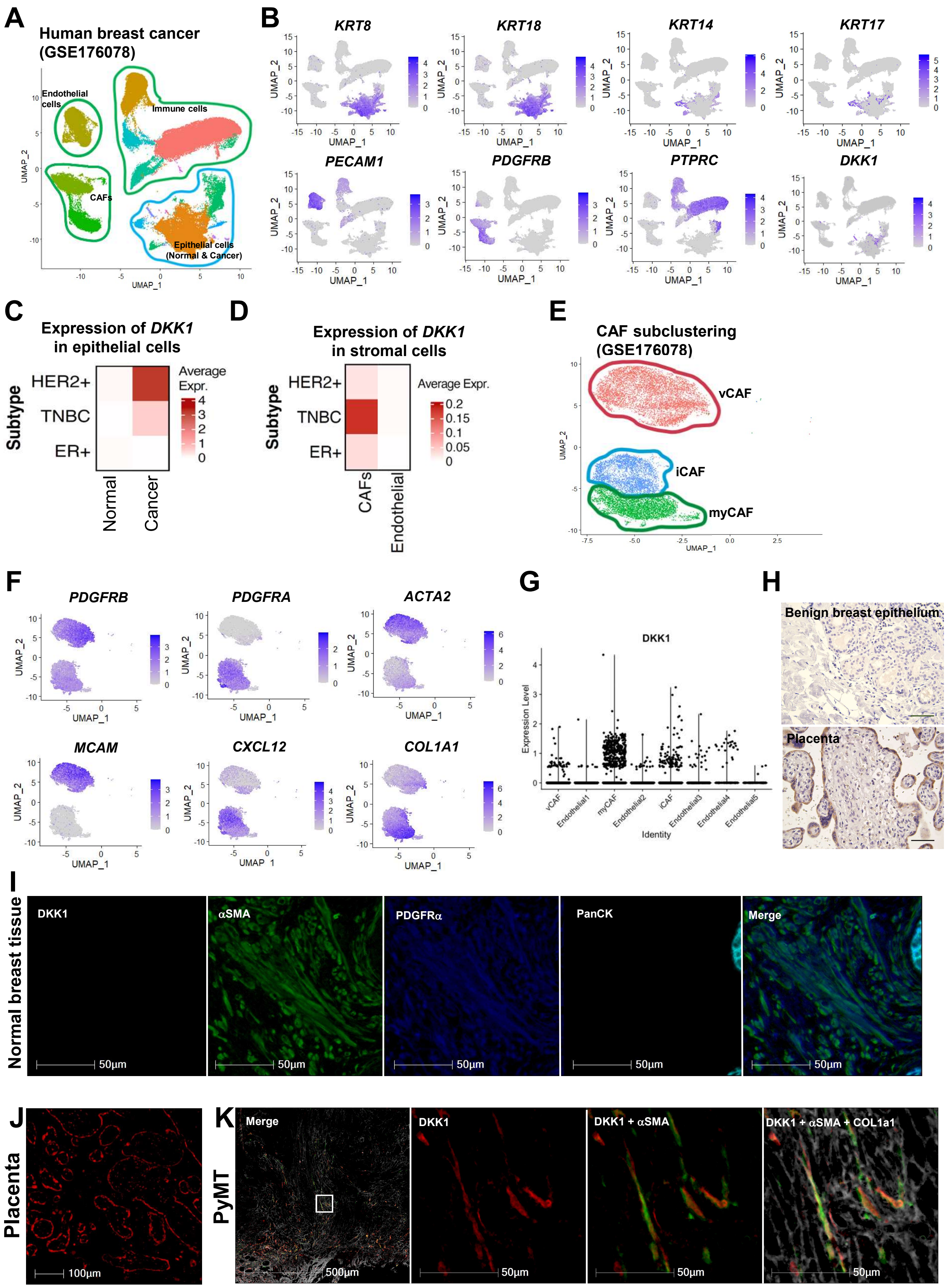

(A, B) UMAP visualization of annotated cell populations in human breast cancer (GSE176078). (C) Heatmap visualization of *DKK1* expression in normal and human breast cancer epithelial cells. (D) Heatmap visualization of *DKK1* expression in the stromal compartment of $HER2^+$, triple-negative, and ER^+ human breast cancer subtypes. (E, F) UMAP visualization of CAF subset clusters. (G) Violin plot of *DKK1* expression in stromal populations. (H) Immunohistochemistry images of benign breast epithelium (top) and placenta (bottom) stained with anti-DKK1 antibody developed with DAB chromogen (brown) and hematoxylin (blue). (I) Multiplex immunohistochemistry (mIHC) of human terminal duct lobular unit in normal breast tissue stained for DKK1 (red), αSMA (green), PDGFRα (blue) and panCK (cyan) (n=3). (J) mIHC of human placenta stained for DKK1 (red) (n=4). (K) mIHC of orthotopic PyMT breast tumors stained for DKK1 (red), αSMA (green), and COL1a1 (white) (n=5).

Supplementary Figure 3.

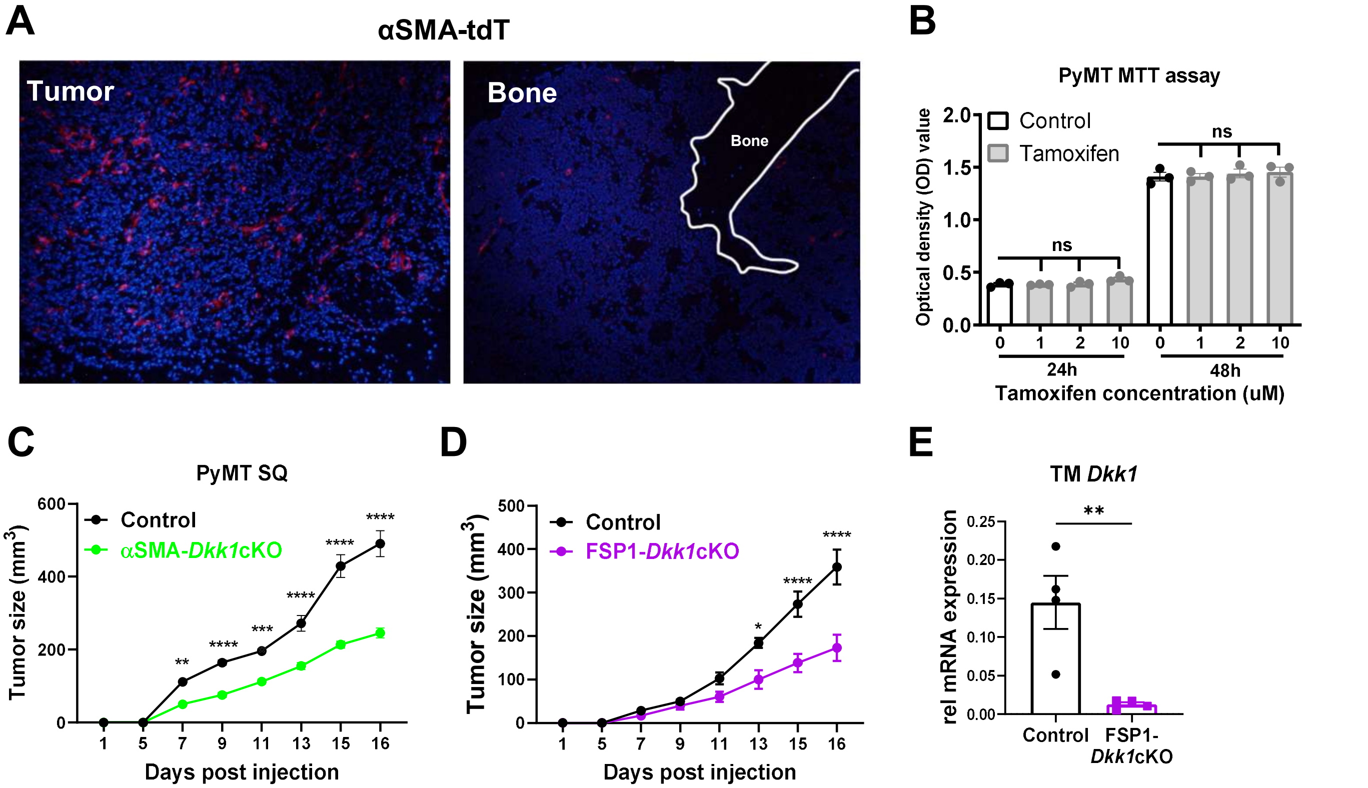

(A) Immunofluorescence analysis of tdT⁺ cells (red) and total cells stained with DAPI (blue) in orthotopic PyMT tumors and bones from 12 weeks old αSMA-tdT mice, receiving tamoxifen at time of tumor inoculation. Trabecular bone depicted by white contour. (B) MTT analysis in PyMT cells stimulated with indicated concentrations of tamoxifen for indicated amount of time. (C) Tumor growth was determined by caliper measurements in 10-12 weeks old, male αSMA-*Dkk1*cKO mice and controls injected with tamoxifen for 5 consecutive days (100mg/kg) starting a day before the subcutaneous (SQ) PyMT tumor inoculation (n=5, 8 mice/group). (D, E) Tumor size and qRT-PCR for *Dkk1* expression in primary tumors from 6-8 weeks old FSP1-*Dkk1*WT (control) and FSP1-*Dkk1*cKO female mice (n=4 mice/group) inoculated with PyMT in the MFP. Experiments in (B) were performed in triplicate. Results represent mean +/- SEM. Ordinary one-way ANOVA followed by Dunnett's multiple-comparison test (B), two-way ANOVA followed by Bonferroni multiple-comparison test (C, D), and unpaired t-test with two-tailed P value for (E) were used to determine significance * $P < 0.05$, ** $P < 0.01$, *** $P < 0.001$, **** $P < 0.0001$.

Supplementary Figure 4.

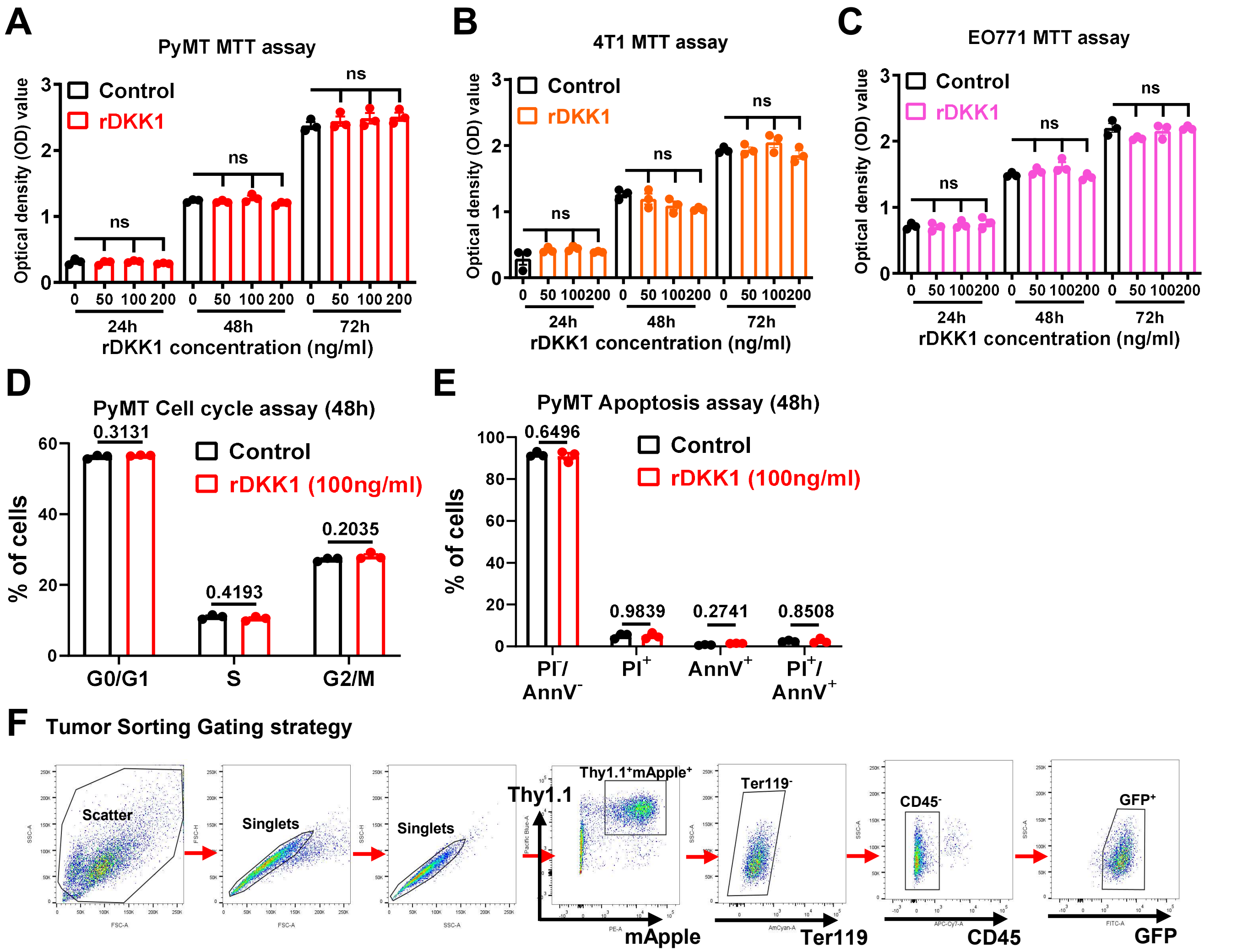

(A-C) MTT analysis in PyMT (A), 4T1 (B), and EO771 (C) tumor cells stimulated with indicated concentrations of recombinant DKK1 (rDKK1). (D, E) cell cycle and apoptosis analyses in PyMT cells stimulated with indicated concentrations of rDKK1 for indicated times. Apoptotic cells were analyzed based on positivity for Propidium Iodide (PI) and Annexin V (AnnV). (F) Gating strategies for sorting PyMT-BO1-GFP-fluc-H2B-mApple-Thy1.1 tumor cells injected in the MFP of WT mice. Experiments in (A-E) were performed in triplicate. Results are shown as mean +/- SEM. Ordinary one-way ANOVA followed by Dunnett's multiple-comparison test (A-C) and unpaired t-test with two-tailed P value (D, E) were used to determine significance .

Supplementary Figure 5.

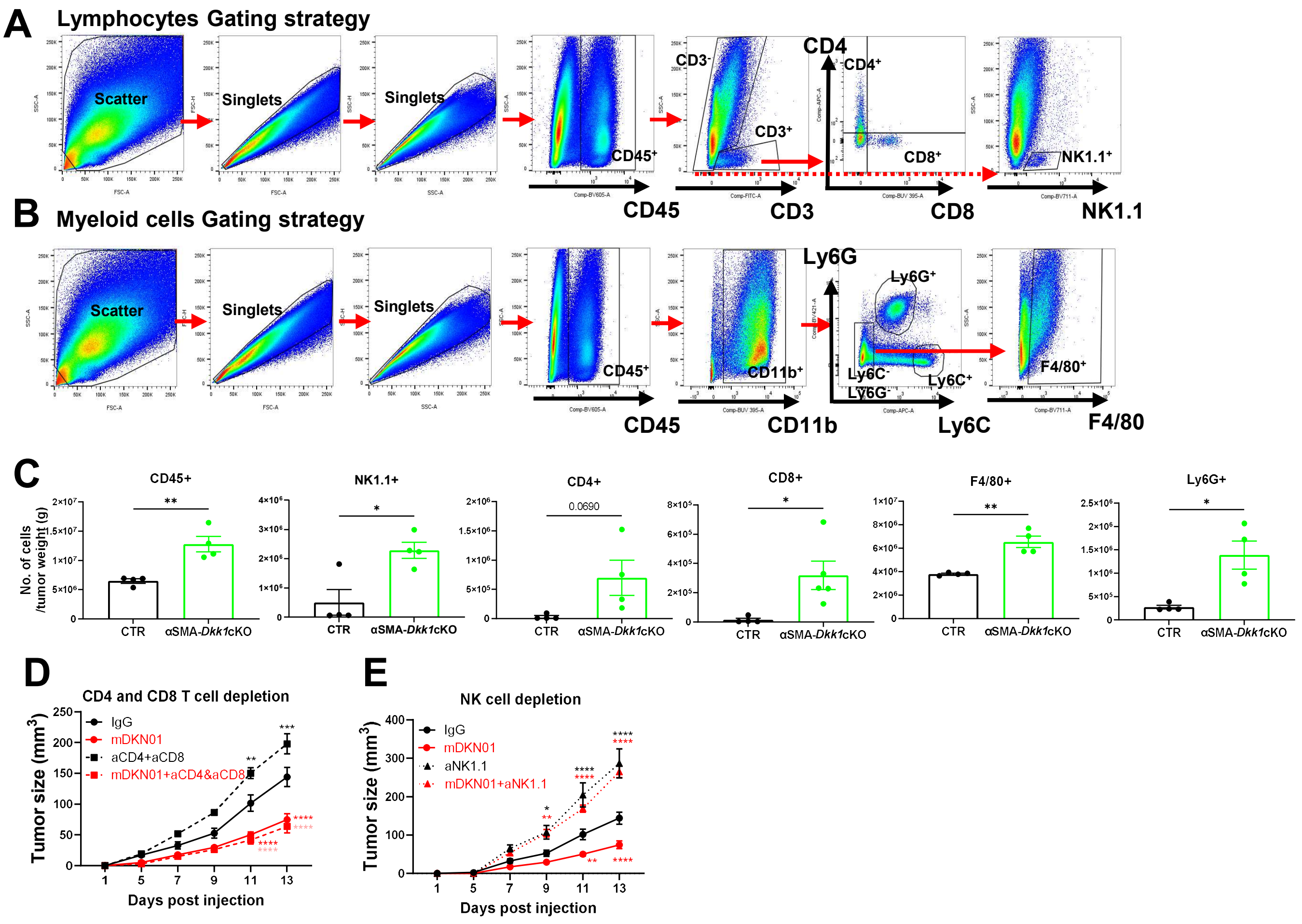

(A, B) Gating strategies for profiling lymphocytes (A) and myeloid cells (B) in PyMT orthotopic tumors from WT mice. (C) FACS analysis of tumor infiltrating $CD45^+$ immune cells, NK cells, T cell and myeloid subsets per gram of orthotopic PyMT tumor isolated from αSMA-*Dkk1*WT (control, CTR) or αSMA-*Dkk1*cKO mice (n=4/group). (D, E) PyMT orthotopic tumor growth determined by caliper measurements in 6-8 weeks WT female mice treated with mDKN01 (10mg/kg) or control IgG antibody every other day along with anti-CD4 and anti-CD8 (D) or anti-NK1.1 (E) (n=4-9 mice/group). Results are shown as mean +/- SEM. Unpaired t-test with two-tailed P value (C) and two-way ANOVA followed by Bonferroni multiple-comparison test (D, E) were used to determine significance. * P < 0.05, ** P < 0.01, *** P < 0.001, **** P < 0.0001.

Supplementary Figure 6.

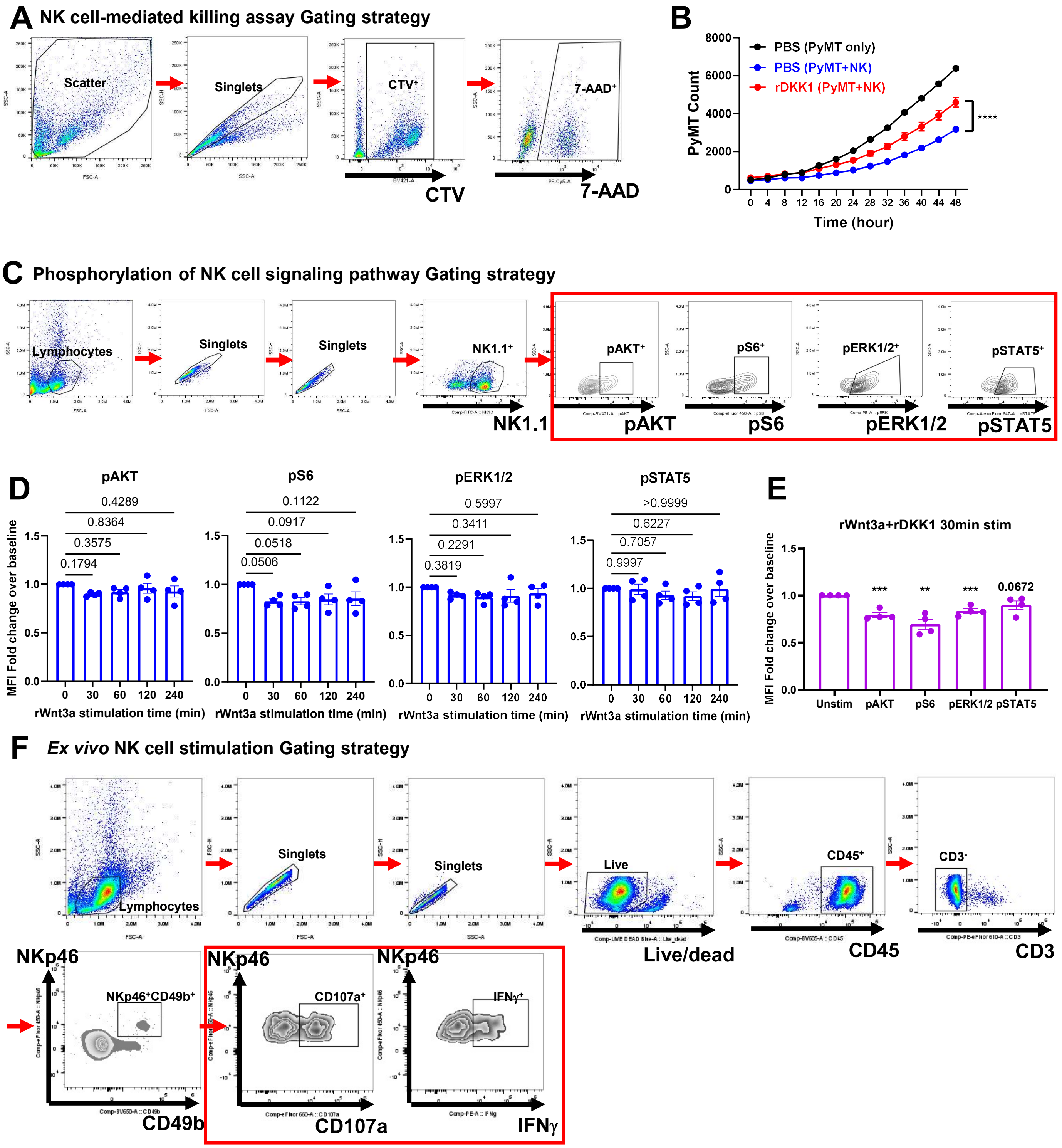

(A) Gating strategy for analyzing NK cell-mediated percent specific killing of target cells (7-AAD$^+$ cells). (B) mApple$^+$ PyMT-BO1 target cell number surviving NK cell-mediated killing in the presence of PBS or rDKK1 analyzed over indicated time. (C) Gating strategy for analyzing phosphorylated proteins by intracellular FACS staining in NK cells isolated from the spleen of *Rag1*$^{-/-}$ mice. (D, E) Fold changes from baseline of mean fluorescence intensity (MFI) measurements of phosphorylated AKT, S6, ERK1/2, and STAT5 in NK cells from the spleen of *Rag1*$^{-/-}$ mice (n=4), following stimulation with rWnt3a (100ng/ml) for indicated time (D) or with rWnt3a (100ng/ml)+rDKK1 (200ng/ml) for 30 min (E). (F) Gating strategy for analyzing CD107a$^+$ and IFNγ$^+$ NK cells in PyMT tumor mass following ex vivo stimulation with anti-NK1.1 antibody and IL12 + IL15, respectively. Experiments in (B) were performed in triplicate. Two-way ANOVA followed by Bonferroni multiple-comparison test (B), ordinary one-way ANOVA followed by Dunnett's multiple-comparison test (D), and unpaired t-test with two-tailed P value (E) were used to determine significance ** P < 0.01, *** P < 0.001, **** P < 0.0001.

Supplementary Figure 7.

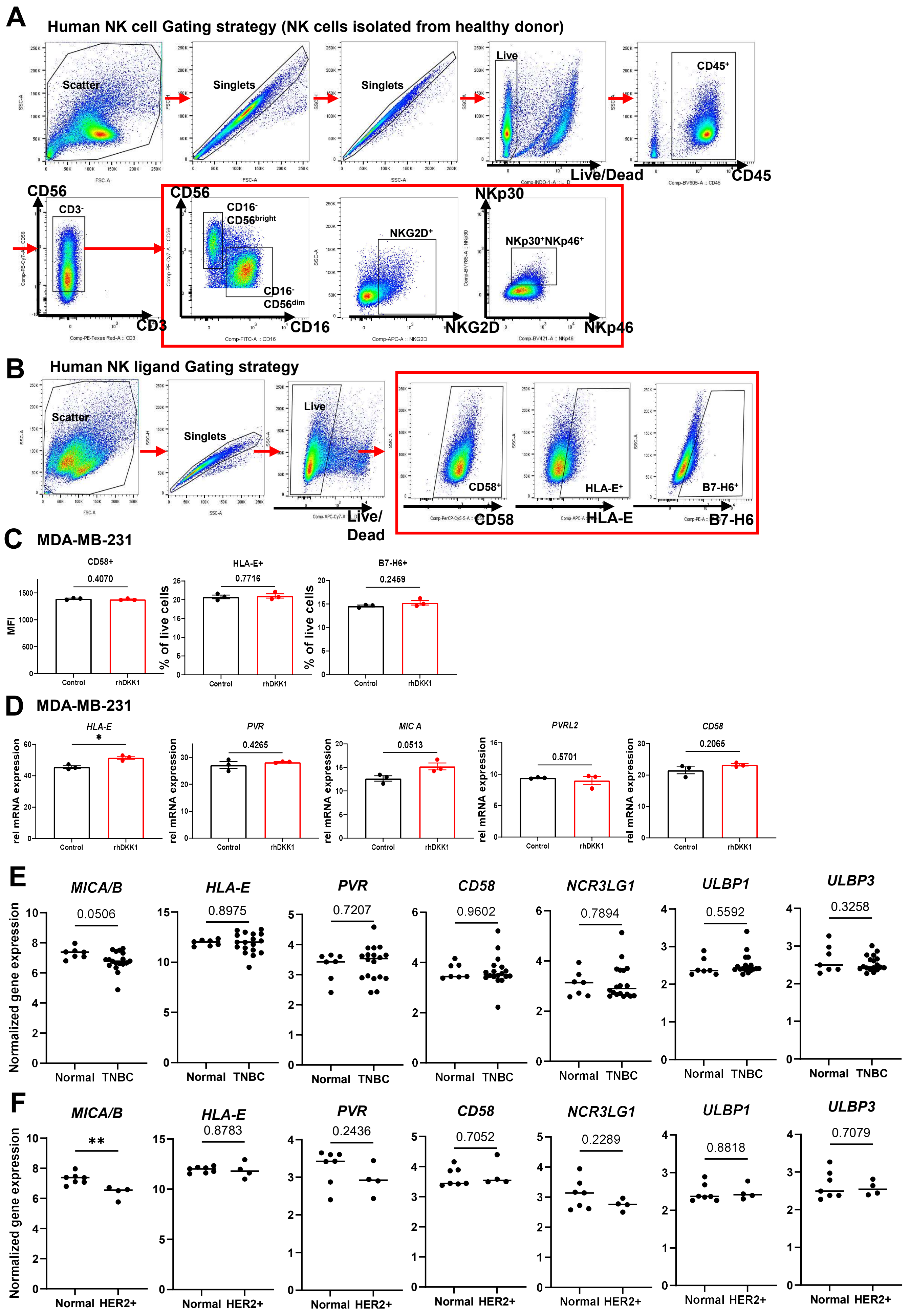

G

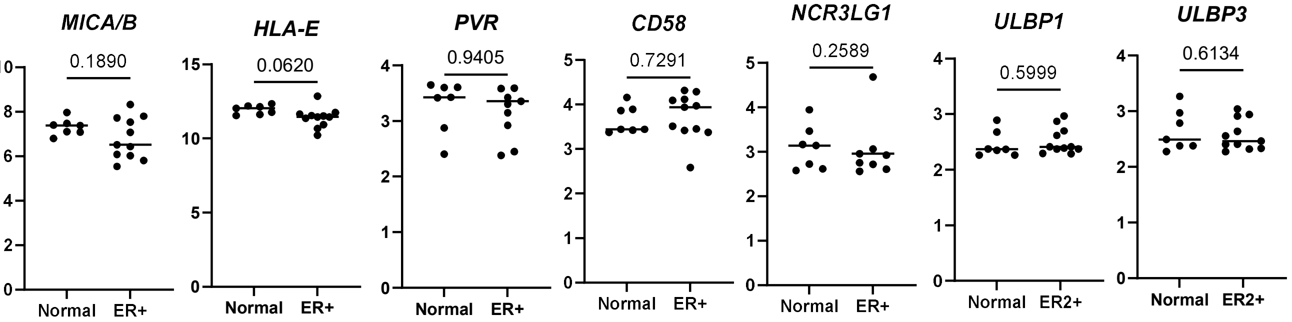

(A, B) Gating strategies for profiling human NK cells isolated from healthy donor peripheral blood (A) and NK cell ligand expression on MDA-MB-231 cells (B). (C) Quantification of NK ligand expression on MDA-MB-231 cells measured by flow cytometry after 4 hours of incubation with rhDKK1 (200ng/ml). (D) qRT-PCR for NK cell ligand expression in MDA-MB-231 cells after stimulation with rhDKK1 (200ng/ml) for 24 hours. (E-G) Normalized gene expression of NK cell ligands in human normal breast tissues versus triple-negative breast cancer (E), $HER2^+$ breast cancer (F), ER^+ breast cancer (G) (GSE3744). Experiments in (C, D) were performed in triplicate. Unpaired t-test with two-tailed P value (C-G) was used to determine significance * $P < 0.05$, ** $P < 0.01$.

Supplementary Figure 8.

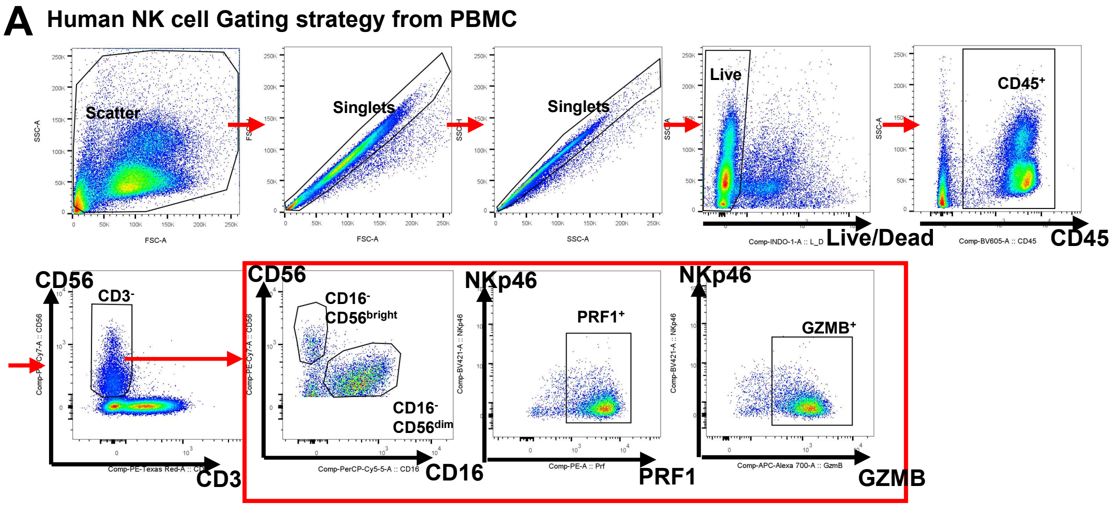

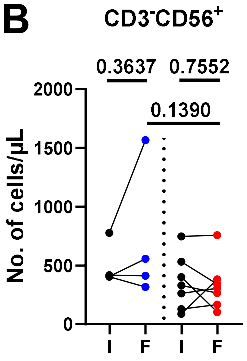

(A) Gating strategy for profiling human NK cells from peripheral blood mononuclear cells (PBMC) of breast cancer patients. (B) Number of NK cells in blood in patients with regressive/stable (blue) versus progressive bone metastases (red) from initial diagnosis (abbreviated as I) and follow-up visits (abbreviated as F). Unpaired t-test and paired t-test with two-tailed P value were used to determine significance.

Supplementary Figure 9

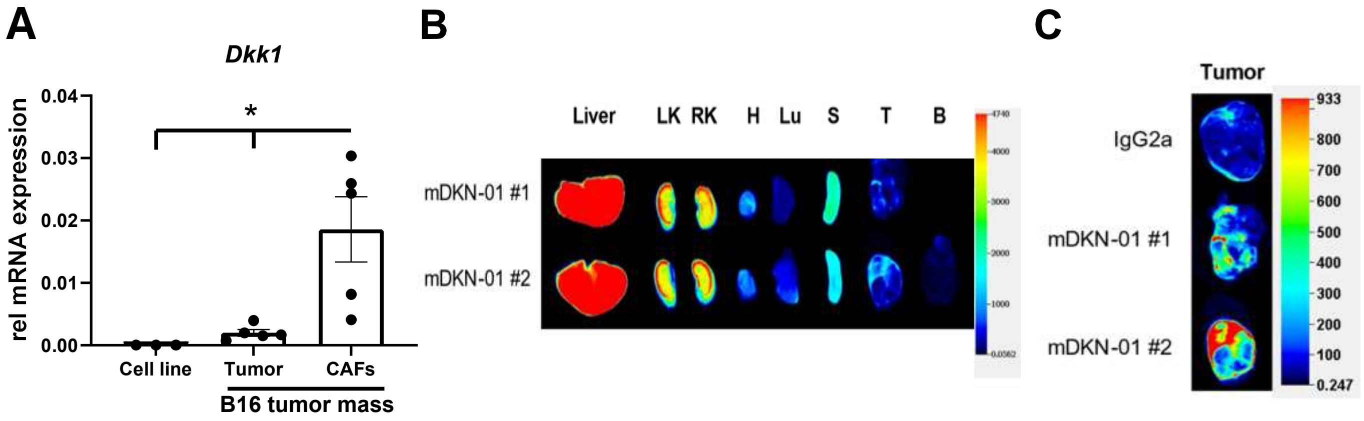

(A) *Dkk1* expression by qRT-PCR in the B16 cell line (n=3), tumor cells and CAFs isolated from the subcutaneous B16-F10 tumors in 6-8 weeks old female C57BL/6 WT mice (n=5). (B, C) The biodistribution of infrared (IR)-dye conjugated mDKN01 or IgG2a antibody was evaluated in B16 tumor-bearing mice. (B, C) The liver, kidneys (left LK and right RK), heart (H), lungs (Lu), spleen (S), tumor mass (T) and brain (B) were subsequently imaged using the Licor Odyssey Clx. Results represent mean +/- SEM. Unpaired t-test with two-tailed P value was used to determine significance. *P<0.05
