# Supplementary material for: Stroma-derived Dickkopf-1 contributes to the suppression of NK cell cytotoxicity in breast cancer
Source: Nat Commun. 2025 Jan 30;16:1183. doi: 10.1038/s41467-025-56420-w (PMC11782527; doi:10.1038/s41467-025-56420-w)
Supplement: Supplementary file 2 — Reporting Summary [file 41467_2025_56420_MOESM2_ESM.pdf]

Corresponding author(s): Roberta Faccio

Last updated by author(s): Dec 20, 2024

Reporting Summary

Nature Portfolio wishes to improve the reproducibility of the work that we publish. This form provides structure for consistency and transparency in reporting. For further information on Nature Portfolio policies, see our Editorial Policies and the Editorial Policy Checklist.

Statistics

For all statistical analyses, confirm that the following items are present in the figure legend, table legend, main text, or Methods section.

n/a	Confirmed	
☐	☒	The exact sample size (*n*) for each experimental group/condition, given as a discrete number and unit of measurement
☐	☒	A statement on whether measurements were taken from distinct samples or whether the same sample was measured repeatedly
☐	☒	The statistical test(s) used AND whether they are one- or two-sided<br>*Only common tests should be described solely by name; describe more complex techniques in the Methods section.*
☒	☐	A description of all covariates tested
☐	☒	A description of any assumptions or corrections, such as tests of normality and adjustment for multiple comparisons
☐	☒	A full description of the statistical parameters including central tendency (e.g. means) or other basic estimates (e.g. regression coefficient) AND variation (e.g. standard deviation) or associated estimates of uncertainty (e.g. confidence intervals)
☐	☒	For null hypothesis testing, the test statistic (e.g. *F*, *t*, *r*) with confidence intervals, effect sizes, degrees of freedom and *P* value noted<br>*Give P values as exact values whenever suitable.*
☒	☐	For Bayesian analysis, information on the choice of priors and Markov chain Monte Carlo settings
☒	☐	For hierarchical and complex designs, identification of the appropriate level for tests and full reporting of outcomes
☒	☐	Estimates of effect sizes (e.g. Cohen's *d*, Pearson's *r*), indicating how they were calculated

Our web collection on statistics for biologists contains articles on many of the points above.

Software and code

Policy information about availability of computer code

Data collection	BD FACSDiva v9.0 or CYTEK SpectroFlo v3.3.0 for FACS, NIS-Elements software (Nikon, ver.5.21.00) for confocal microscope, IncuCyte Live-cell Analysis system (Satorius) for killing assay.
Data analysis	Data was analyzed in GraphPad Prism (version 10). All IHC analyses were performed on the HALO image analysis platform (Indica Labs, Deconvolution v1.1.1, Multiplex IHC v.3.2.3 algorithms). Bioluminescence photon flux (photons per second) was analyzed via measurements of region of interest (fixed region of interest over the whole body, or hindlimb) using Living Image 3.2 (Caliper Life Sciences). Bulk RNA sequencing data was analyzed using the Partek Flow software (Partek Inc., St. Louis, MO). Flow cytometry data was analyzed with FlowJo 10.9.0 software (Tree Star). Images were analyzed with NIS-Elements software (Nikon, ver.5.21.00) and ImageJ. Killing assay was analyzed with Incucyte 2022A Rev1 (Satorius).

For manuscripts utilizing custom algorithms or software that are central to the research but not yet described in published literature, software must be made available to editors and reviewers. We strongly encourage code deposition in a community repository (e.g. GitHub). See the Nature Portfolio guidelines for submitting code & software for further information.

Data

Policy information about availability of data

All manuscripts must include a data availability statement. This statement should provide the following information, where applicable:

- Accession codes, unique identifiers, or web links for publicly available datasets
- A description of any restrictions on data availability
- For clinical datasets or third party data, please ensure that the statement adheres to our policy

The bulk RNA sequencing data generated in this study have been deposited in NCBI's Gene Expression Omnibus63 and are accessible through GEO Series accession number GSE 262733 [https://www.ncbi.nlm.nih.gov/geo/query/acc.cgi?&acc=GSE262733]. Previously published data are accessible through GEO Series accession number (GSE3644 [https://www.ncbi.nlm.nih.gov/geo/query/acc.cgi?acc=GSE3744], GSE8977 [https://www.ncbi.nlm.nih.gov/geo/query/acc.cgi?acc=GSE8977], GSE176078 [https://www.ncbi.nlm.nih.gov/geo/query/acc.cgi?acc=GSE176078]). The remaining data are available within the Article, Supplementary Information or Source data file.

Research involving human participants, their data, or biological material

Policy information about studies with human participants or human data. See also policy information about sex, gender (identity/presentation), and sexual orientation and race, ethnicity and racism.

Reporting on sex and gender	Women with a confirmed diagnosis of breast cancer were eligible for this study.
Reporting on race, ethnicity, or other socially relevant groupings	Patients were not excluded from participation in this study based on race, ethnicity, or any other socially relevant construct, as cancer affects women of all races/ethnicities. All race and ethnicity data collected was self-reported by each enrolling patient. Data presented in this manuscript was blinded to race, ethnicity, and all other socially relevant constructs.
Population characteristics	All patients determined to be eligible for this study, were separated into two cohorts based on the stage of their disease: early stage or metastatic. For analysis purposes, prior diagnoses and treatment history was obtained.
Recruitment	Patients at the Washington University in St. Louis School of Medicine were recruited specifically from the Siteman Cancer Center outpatient population or patient referrals by our community oncologists to the Principal Investigator (PI) and co-investigators. The recruitment process did not involve any restrictions based on social or demographic factors including age or ethnic characteristics of the subject population. The clinical information of the enrolled patients, including age, timeline of visits, sites of metastatic dissemination and treatment histories, was reviewed and documented by the breast oncologist C.X.M.
Ethics oversight	The study protocol was approved by the Washington University in St. Louis School of Medicine Institutional Review Board (IRB) as part of the Human Research Protection Office (HRPO). All participants gave written informed consent under the IRB-approved protocol prior to inclusion in the study, including access of archival tumor tissue for research.

Note that full information on the approval of the study protocol must also be provided in the manuscript.

Field-specific reporting

Please select the one below that is the best fit for your research. If you are not sure, read the appropriate sections before making your selection.

☒ Life sciences ☐ Behavioural & social sciences ☐ Ecological, evolutionary & environmental sciences

For a reference copy of the document with all sections, see nature.com/documents/nr-reporting-summary-flat.pdf

Life sciences study design

All studies must disclose on these points even when the disclosure is negative.

Sample size	Sample size was calculated based on our previous published data and preliminary experiments.
Data exclusions	Outlier test was performed to determine whether any data point should have been excluded. (P<0.05 (two-sided))
Replication	In vitro experiments include technical and biological triplicates and were performed at least 3 times. In vivo experiments were done with at least 4 to 9 mice per group (the number of mice used for each experiment is specified in the figure legends) and at least 3 independent experiments were performed. All attempts at replication were successful.
Randomization	Mice were randomized before treatment.
Blinding	Blinding was not possible due to the repeated measurements of tumor progression over time and delivery of specific treatments.

Reporting for specific materials, systems and methods

We require information from authors about some types of materials, experimental systems and methods used in many studies. Here, indicate whether each material, system or method listed is relevant to your study. If you are not sure if a list item applies to your research, read the appropriate section before selecting a response.

Materials & experimental systems

n/a	Involved in the study
☐	☒ Antibodies
☐	☒ Eukaryotic cell lines
☒	☐ Palaeontology and archaeology
☐	☒ Animals and other organisms
☐	☒ Clinical data
☒	☐ Dual use research of concern
☒	☐ Plants

Methods

n/a	Involved in the study
☒	☐ ChIP-seq
☐	☒ Flow cytometry
☒	☐ MRI-based neuroimaging

Antibodies

Antibodies used

<IHC/IF>
aSMA (Abcam, Cat# ab5694, 1:1500 (Human), 1:200 (Mouse)), COL1a1 (Cell signaling, Cat# 72026, 1:100 (Mouse)), COL14a1 (Cell signaling, Cat# 61964, 1:200 (Human)), DKK1 (Proteintech, Cat# 21112-1-AP, 1:100 (Human), 1:3000 (Mouse)), PDGFRa (Cell signaling, Cat# 5241, 1:200 (Human)), PanCK (Novus, Cat# NBP2-29429, 1:1000 (Human)), anti-rabbit-Alexa 488 (Abcam, Cat# ab150077, 1:1000), F-actin-Alexa488 (Invitrogen, Cat# A12379, 1:200)

<Flow cytometry; Anti-mouse>
CD3e-FTIC (Biolegend, Cat# 100306, Clone 17A2, 1:400), CD4-APC (BD Pharmingen, Cat# 561091, Clone RM4-5, 1:200), CD8a-BUV395 (BD Horizon, Cat# 565968, Clone 53-6.7, 1:200), CD11b-BUV395 (BD Biosciences, Cat# 565976, Clone M1/70, 1:400), CD16/32 (Biolegend, Cat# 101302, Clone 93, 1:500), CD45-APC Cy7 (Biolegend, Cat# 103116, Clone 30-F11, 1:400), CD45-BV605 (Biolegend, Cat# 103140, Clone 30-F11, 1:400), CD107a-eFlour660 (ThermoFisher, Cat# 50-1071-82, Clone 1D4B, 1:200), F4/80-BV711 (Biolegend, Cat# 123147, Clone BM8, 1:100), IFNg-BV750 (Biolegend, Cat# 505865, Clone XMG1.2, 1:50), Ly6C-APC (Biolegend, Cat# 128016, Clone HK1.4, 1:400), Ly6G-BV421 (BD Biosciences, Cat# 562737, Clone 1A8, 1:400), NK1.1-BV711 (Biolegend, Cat# 108745, Clone PK136, 1:200), pAKT-BV421 (BD phosflow, Cat# 562599, Clone M89-61, 1:20), pERK1/2-PE (BD phosflow, Cat# 612566, Clone 20A, 1:10), pS6-eFlour450 (BD phosflow, Cat# 561457, Clone N7-548, 1:20), pSTAT5-Alexa Fluor647 (BD phosflow, Cat# 612599, Clone 47/Stat5, 1:20), Ter119-BV605 (Biolegend, Cat# 116239, Clone TER-119, 1:200), CD90.1-eFlour450 (ThermoFisher, Cat# 48-0900-82, Clone HIS51, 1:200)

<Flow cytometry; Anti-human>
CD3-ECD (Beckman Coulter, Cat# A07748, Clone UCHT1, 1:50), CD16-PerCP Cy5.5 (BD Pharmingen, Cat# 560717, Clone 3G8, 1:200), CD45-BV605 (Biolegend, Cat# 304042, Clone 2D1, 1:20), CD56-PE Cy7 (Beckman Coulter, Cat# A51078, Clone N901, 1:100), CD58-PerCP Cy5.5 (Biolegend, Cat# 330914, Clone TS2/9, 1:50), B7-H6-PE (R&D systems, Cat# FAB7144P, Clone 875001, 1:20), GzmB-AF700 (BD Pharmingen, Cat# 560213, Clone GB11, 1:50), HLA-E-APC (Biolegend, Cat# 342606, Clone 3D12, 1:20), NKG2D-APC (Invitrogen, Cat# 17-5878-42, Clone 1D11, 1:20), NKp30-BV785 (Biolegend, Cat# 325229, Clone P30-15, 1:20), NKp46-BV421 (BD Horizon, Cat# 564065, Clone 9E2, 1:20), PRF1-PE (Biolegend, Cat# 308106, Clone dG9, 1:50)

<Neutralization; Anti-mouse>
IgG isotype control (for anti-DKK1 control, Absolute antibody, FcR incompetent construct (D265A), Clone 4-4-20), anti-DKK1 (mDKN01, Leap therapeutics, Clone 4-4-20), anti-CD4 (BioXCell, Cat# BE0003, Clone GK1.5), anti-CD8a (BioXCell, Cat# BE0061, Clone 2.43), anti-NK1.1 (Leinco Technologies, Inc., Cat# N268, Clone PK136)

Validation

Validation of commercial antibodies used in this study was done by the manufacturer, and details of these validation efforts are described on the manufacturer's website.
<IHC/IF>
aSMA (https://www.abcam.com/en-us/products/primary-antibodies/alpha-smooth-muscle-actin-antibody-ab5694?srsltid=AfmBOoqTmDTB5YMPYnC6WRRgHkg3g2S66BJ_LTzBxziQ4R441UYQaSQb#tab=datasheet), COL1a1 (https://www.cellsignal.com/products/primary-antibodies/col1a1-e8f4l-xp-rabbit-mab/72026?srsltid=AfmBOor7QKuFdopHU5XbvPr9XwJu85I1RAsOHaVHG6Eabz_IuKCC0Kwl), COL14a1 (https://www.cellsignal.com/products/primary-antibodies/col14a1-e5w8s-rabbit-mab/61964), DKK1 (https://www.ptglab.com/products/DKK1-Antibody-21112-1-AP.htm?srsltid=AfmBOoqunXzqe0KrSJVYl6rv37XgiQAfrZm03MFX_L7Uh2rQhKhbbJHq), PDGFRa (https://www.cellsignal.com/products/primary-antibodies/pdgf-receptor-a-d13c6-xp-rabbit-mab/5241), PanCK (https://www.novusbio.com/products/cytokeratin-pan-antibody-ae-1-ae-3_nbp2-29429?srsltid=AfmBOooV8VseKjx81OkTgxqvtWsz70zwCdACQ0luvxQwREFmGKY0nEXc#reviews-publications). F-actin-Alexa488 (https://www.thermofisher.com/order/catalog/product/A12379)

<Flow cytometry; Anti-mouse>
CD3e-FITC (https://www.biolegend.com/en-us/products/fitc-anti-mouse-cd3epsilon-antibody-23), CD4-APC (https://www.bdbiosciences.com/en-us/products/reagents/flow-cytometry-reagents/research-reagents/single-color-antibodies-ruo/apc-rat-anti-mouse-cd4.561091?tab=product_details), CD8a-BUV395 (https://www.bdbiosciences.com/en-us/products/reagents/flow-cytometry-reagents/research-reagents/single-color-antibodies-ruo/buv395-rat-anti-mouse-cd8a.565968?tab=product_details), CD11b-BUV395 (https://www.bdbiosciences.com/en-us/products/reagents/flow-cytometry-reagents/research-reagents/single-color-

antibodies-ruo/buv395-rat-anti-cd11b.565976?tab=product_details), CD16/32 (https://www.biolegend.com/en-us/products/purified-anti-mouse-cd16-32-antibody-190), CD45-APC Cy7 (https://www.biolegend.com/en-us/products/apc-cyanine7-anti-mouse-cd45-antibody-2530), CD45-BV605 (https://www.biolegend.com/en-us/products/brilliant-violet-605-anti-mouse-cd45-antibody-8721), CD107a-eFlour660 (https://www.thermofisher.com/antibody/product/CD107a-LAMP-1-Antibody-clone-eBio1D4B-1D4B-Monoclonal/50-1071-82), F4/80-BV711 (https://www.biolegend.com/en-us/products/brilliant-violet-711-anti-mouse-f4-80-antibody-10705), IFNg-BV750 (https://www.biolegend.com/en-us/products/brilliant-violet-750-anti-mouse-ifn-gamma-antibody-24137), Ly6C-APC (https://www.biolegend.com/en-us/products/apc-anti-mouse-ly-6c-antibody-6047), Ly6G-BV421 (https://www.bdbiosciences.com/en-us/products/reagents/flow-cytometry-reagents/research-reagents/single-color-antibodies-ruo/bv421-rat-anti-mouse-ly-6g.562737?tab=product_details), NK1.1-BV711 (https://www.biolegend.com/en-us/products/brilliant-violet-711-anti-mouse-nk-1-1-antibody-9576), pAKT-BV421 (https://www.bdbiosciences.com/en-us/products/reagents/flow-cytometry-reagents/research-reagents/single-color-antibodies-ruo/bv421-mouse-anti-akt-ps473.562599?tab=product_details), pERK1/2-PE (https://www.bdbiosciences.com/en-us/products/reagents/flow-cytometry-reagents/research-reagents/single-color-antibodies-ruo/pe-mouse-anti-erk1-2-pt202-py204.612566?tab=product_details), pS6-eFlour450 (https://www.bdbiosciences.com/en-us/products/reagents/flow-cytometry-reagents/research-reagents/single-color-antibodies-ruo/v450-mouse-anti-s6-ps235-ps236.561457), pSTAT5-Alexa Fluor647 (https://www.bdbiosciences.com/en-us/products/reagents/flow-cytometry-reagents/research-reagents/single-color-antibodies-ruo/alexa-fluor-647-mouse-anti-stat5-py694.612599?tab=product_details), Ter119-BV605 (https://www.biolegend.com/en-us/products/brilliant-violet-605-anti-mouse-ter-119-erythroid-cells-antibody-8839), CD90.1-eFlour450 (https://www.thermofisher.com/antibody/product/CD90-1-Thy-1-1-Antibody-clone-HIS51-Monoclonal/48-0900-82)

<Flow cytometry; Anti-human>
CD3-ECD (https://www.beckman.kr/reagents/coulter-flow-cytometry/antibodies-and-kits/single-color-antibodies/cd3/a07748), CD16-PerCP Cy5.5 (https://www.bdbiosciences.com/en-us/products/reagents/flow-cytometry-reagents/research-reagents/single-color-antibodies-ruo/percp-cy-5-5-mouse-anti-human-cd16.560717), CD45-BV605 (https://www.biolegend.com/en-us/products/brilliant-violet-605-anti-human-cd45-antibody-8521), CD56-PE Cy7 (https://www.beckman.kr/reagents/coulter-flow-cytometry/antibodies-and-kits/single-color-antibodies/cd56/a51078), CD58-PerCP Cy5.5 (https://www.biolegend.com/en-us/products/percp-cyanine5-5-anti-human-cd58-lfa-3-antibody-14107), B7-H6-PE (https://www.rndsystems.com/products/human-b7-h6-pe-conjugated-antibody-875001_fab7144p), GzmB-AF700 (https://www.bdbiosciences.com/en-us/products/reagents/flow-cytometry-reagents/research-reagents/single-color-antibodies-ruo/alexa-fluor-700-mouse-anti-human-granzyme-b.560213?tab=product_details), HLA-E-APC (https://www.biolegend.com/en-us/products/apc-anti-human-hla-e-antibody-10760), NKG2D-APC (https://www.thermofisher.com/antibody/product/CD314-NKG2D-Antibody-clone-1D11-Monoclonal/17-5878-42), NKp30-BV785 (https://www.biolegend.com/en-us/products/brilliant-violet-785-anti-human-cd337-nkp30-antibody-17800), NKp46-BV421 (https://www.bdbiosciences.com/en-us/products/reagents/flow-cytometry-reagents/research-reagents/single-color-antibodies-ruo/bv421-mouse-anti-human-cd335-nkp46.564065), PRF1-PE (https://www.biolegend.com/en-us/products/pe-anti-human-perforin-antibody-885)

<Neutralization; Anti-mouse>
anti-CD4 (https://bioxcell.com/invivomab-anti-mouse-cd4-be0003-1?queryID=6d664e7480bc3adce365b65d563e4bf8&objectID=30483&indexName=bioxcell_live_default_products), anti-CD8a (https://bioxcell.com/invivomab-anti-mouse-cd8-alpha-be0061), anti-NK1.1 (https://www.leinco.com/p/anti-mouse-nk1-1-purified-functional-grade-platinum/)

Specific cell depletion was validated by FACS analysis. Anti-DKK1 antibody was validated in DKK1 knock out mouse model. Antibodies against activated immune cell markers were validated by comparing unstimulated and stimulated immune cells with proper activating reagents. Antibodies against surface markers were validated using different clones of antibodies.

Eukaryotic cell lines

Policy information about cell lines and Sex and Gender in Research

Cell line source(s)	Polyoma middle tumor-antigen murine mammary tumor cells (PyMT, C57BL/6; generously provided by David DeNardo, Washington University in St. Louis, MO), mCherry-conjugated PyMT (PyMT-mCherry; generously provided by David DeNardo, Washington University in St. Louis, MO), PyMT-derivative PyMT-BO1 conjugated with firefly luciferase (PyMT-BO1-fluc; generously provided by Katherine Weilbaecher, Washington University in St. Louis, MO), and H2B-mApple and Thy1.1 conjugated PyMT-BO1 (PyMT-BO1-GFP-fluc-H2B-mApple-Thy1.1; generously provided by Sheila Stewart, Washington University in St. Louis, MO), EO771 murine mammary tumor cells (EO771-fluc, C57BL/6; generously provided by Sheila Stewart, Washington University in St. Louis, MO), 4T1 murine mammary tumor cells (4T1-GFP-fluc, BALB/c; generously provided by David Piwnica-Worms, The University of Texas MD Anderson, Houston TX), T47D human breast cancer cells (provided by ATCC, HTB-133), and MDA-MB-231 human breast cancer cells (provided by ATCC, HTB-26). K562 cell line (provided by ATCC, CRL-3343)
Authentication	No cell line authentication was performed beyond information from suppliers.
Mycoplasma contamination	All cell lines tested negative for Mycoplasma contamination during routine evaluations. Aliquots for each cell line were used for maximum 1 month after initial thaw.
Commonly misidentified lines (See ICLAC register)	No commonly misidentified cell lines were used.

Animals and other research organisms

Policy information about studies involving animals; ARRIVE guidelines recommended for reporting animal research, and Sex and Gender in Research

Laboratory animals	Because the breast cancer cell lines used in this study were obtained from female mice, we have restricted our analyses to females.

Laboratory animals	Female wild-type (WT) C57BL/6 (The Jackson Laboratory (JAX), #000664), WT BALB/c (JAX #000651), B6(Cg)-Tyrc-2J/J (albino C57BL/6, JAX #000058), B6.129S7-Rag1tm1Mom/J (RAG1 KO, JAX #002216), Nu/J (nude, JAX #002019), NOD.Cg-PrkdcscidIl2rgtm1Wjl/SzJ (NSG, JAX #005557), C57BL/6-Prf1tm1Sdz/J (Prf1-/-, JAX #002407), Sp7 Cre (Sp7-tTA, tetO-EGFP/Cre, JAX #006361) mice were purchased from The Jackson Laboratory. Mice arrived at 4–6 weeks of age and were allowed to recover from shipping stress and acclimatize to the new environment for at least 2 weeks before use in experiments. aSMACreERT2 transgenic mice were a generous gift from Dr. Ivo Kalajzic (University of Connecticut Health Center, Farmington, CT). Fsp1 Cre mice were a generous gift from Dr. Regis J. O'Keefe (Washington University, St. Louis, MO). Dkk1 floxed mice were a generous gift from Seppo J. Vainio (University of Oulu, Finland). 6-8 weeks of age female mice used for WT C57BL/6, WT BALB/c, albino C57BL/6, RAG1 KO, nude, NSG, Prf1 KO, Sp7-Dkk1 WT, Sp7-Dkk1 cKO, FSP1-Dkk1 WT, and FSP1-Dkk1 cKO. 8-10 weeks of age male and female aSMA-Dkk1 WT, aSMA-Dkk1 cKO were used. Animals were housed in a pathogen-free animal facility at Washington University (St. Louis, MO) with a 12-hour light/12-hour dark cycle and 20~23°C and 40~60% humidity housing conditions. All experiments were performed according to protocols approved by the Institutional Animal Care and Use Committee at Washington University (Protocol ID: 2022-0315).
Wild animals	The study did not involve wild animals.
Reporting on sex	To study the role of DKK1 in breast cancer, female mice were used. In one experiment, to exclude the possibility that tamoxifen could affect PyMT tumor growth through suppression of estrogen, we used male mice.
Field-collected samples	Human research participants: Women with a confirmed diagnosis of breast cancer were recruited at the Washington University in St. Louis School of Medicine specifically from the Siteman Cancer Center outpatient population or patient referrals by our community oncologists to the Principal Investigator (PI) and co- investigators. The recruitment process did not involve any restrictions based on social or demographic factors including age or ethnic characteristics of the subject population.
Ethics oversight	Mice were used in all experiments according to protocols approved by the Institutional Animal Care and Use Committee at Washington University (Protocol ID: 2022-0315).

Note that full information on the approval of the study protocol must also be provided in the manuscript.

Clinical data

Policy information about clinical studies

All manuscripts should comply with the ICMJE guidelines for publication of clinical research and a completed CONSORT checklist must be included with all submissions.

Clinical trial registration	N/A
Study protocol	IRB ID#: 201102244
Data collection	All participants gave written informed consent under the IRB-approved protocol prior to inclusion in the study, including access of archival tumor tissue for research. Samples were deidentified prior to sharing with collaborators. All studies were conducted in compliance with the Declaration of Helsinki. Patients had diagnosis of estrogen receptor (ER)–positive, HER2-negative breast cancer, stage IV, with bone metastases, prior to first line systemic therapy for metastatic breast cancer or had prior therapy for metastatic breast cancer but met the following criteria: (i) no prior chemotherapy or immune therapy in the past 2 months, (ii) patients currently stable or progressing on hormonal therapy or hormonal therapy combination or starting hormonal therapy or hormonal therapy combination, and (iii) no limitation on the number of prior hormonal therapy or chemotherapy treatments. Radiologic tumor assessment was required within 1 month prior to or after the collection of the baseline blood sample to serve as the baseline tumor assessment. Exclusion criteria included: uncorrected coagulopathy, bleeding tendency, or other conditions that might increase the risk of a biopsy, blood draw, or other procedure; any reason that would make the patient unlikely to comply with study requirements or be incapable of providing appropriate consent (e.g., confusion, infirmity, alcoholism, etc.). Prior history of other invasive malignancies was not an exclusion criterion unless the disease was active and progressing at the time of protocol screening. The clinical information of the enrolled patients, including age, timeline of visits, sites of metastatic dissemination and treatment histories, was reviewed and documented by the breast oncologist C.X.M.
Outcomes	N/A

Plants

Seed stocks	N/A
Novel plant genotypes	N/A
Authentication	N/A

Flow Cytometry

Plots

Confirm that:

☒ The axis labels state the marker and fluorochrome used (e.g. CD4-FITC).

☒ The axis scales are clearly visible. Include numbers along axes only for bottom left plot of group (a 'group' is an analysis of identical markers).

☒ All plots are contour plots with outliers or pseudocolor plots.

☒ A numerical value for number of cells or percentage (with statistics) is provided.

Methodology

Sample preparation	Immediately upon sacrifice, single-cell suspensions were prepared from tumors. In brief, tumor tissues were minced, and then digested with 3.0 mg/ml collagenase A (Roche) and 50 U/ml DNase I (Sigma-Aldrich) in serum free media for 30 min at 37°C. Cells were filtered through 70um nylon strainers (Thermo Fisher Scientific) and washed twice in PBS with 2% FBS. Red blood cells were then removed with red blood cell lysis buffer (Sigma-Aldrich). Cells were washed once, blocked with anti-mouse CD16/CD32 blocker and stained in PBS with 0.5% BSA, 2mM EDTA, and 0.01% NaN3 with the anti–mouse antibodies.
Instrument	Acquisition was performed on a BD LSRFortessa X-20 Cell Analyzer or Cytek Northern Lights.
Software	For acquiring the data, dedicated software DIVA (BD)/ SpectroFlo (Cytek) was used. Data were analyzed with FlowJo 10.9.0 software (Tree Star).
Cell population abundance	After sorting, collected cells were acquired via BD LSRFortessa X-20 Cell Analyzer and confirmed the purity was over 95 percentile.
Gating strategy	FSC-A vs FSC-H gating and SSC-A vs SSC-H gating were utilized to exclude doublets. Then, if applicable, live cells were gated based on the negativity on live/dead dye. Immune cells from CD45 positive gating were analyzed for further profiling. (specified in supplementary gating strategies). For killing assay experiments, CTV positive cells were gated after singlet gating and analyzed for positivity of 7-AAD. For sorting tumor cells, mApple and Thy1.1 double positive populations were selected and excluded Ter119 and CD45 cells based on the negativity.

☒ Tick this box to confirm that a figure exemplifying the gating strategy is provided in the Supplementary Information.
